# A Randomized Controlled Trial Evaluating the Relative Effectiveness of the Multiple Traffic Light and Nutri-Score Front of Package Nutrition Labels

**DOI:** 10.3390/nu11092236

**Published:** 2019-09-17

**Authors:** Eric A. Finkelstein, Felicia Jia Ler Ang, Brett Doble, Wei Han Melvin Wong, Rob M. van Dam

**Affiliations:** 1 Program in Health Services and Systems Research, Duke-NUS Medical School, Singapore 807443, Singapore; felicia.ang@u.duke.nus.edu (F.J.L.A.); brett.doble@duke-nus.edu.sg (B.D.); melvin.wong@duke-nus.edu.sg (W.H.M.W.); 2 Saw Swee Hock School of Public Health, National University of Singapore, Singapore 807443, Singapore; ephrmvd@nus.edu.sg

**Keywords:** front-of-pack labeling, nutrition labeling, food Intake, diet quality, diet, online grocery store, nutri-score, 5-color nutritional label, multiple traffic lights

## Abstract

The objective of this trial was to test two promising front-of-pack nutrition labels, 1) the United Kingdom’s Multiple Traffic Lights (MTL) label and 2) France’s Nutri-Score (NS), relative to a no-label control. We hypothesized that both labels would improve diet quality but NS would be more effective due to its greater simplicity. We tested this hypothesis via an online grocery store using a 3 × 3 crossover (within-person) design with 154 participants. Outcomes assessed via within person regression models include a modified Alternative Healthy Eating Index (AHEI)-2010 (primary), average Nutri-Score, calories purchased, and singular measures of diet quality of purchase orders. Results show that both labels significantly improve modified AHEI scores relative to Control but neither is statistically superior using this measure. NS performed statistically better than MTL and Control based on average Nutri-Score, yet, unlike MTL it did not statistically reduce calories or sugar from beverages. This suggest that NS may be preferred if the goal is to improve overall diet quality but, because calories are clearly displayed on the label, MTL may perform better if the goal is to reduce total energy intake.

## 1. Introduction

All nations have seen a significant upward trend in obesity rates over the past several decades, putting populations at increased risk for weight-related chronic diseases and premature mortality [1,2,3,4,5,6,7,8]. As a result, interventions aimed at encouraging healthier food consumption have been pursued by policy-makers worldwide. Singapore, the country of focus for this study, is no exception. In Singapore, 1 in 3 adults are overweight and 1 in 9 have diabetes [9]. These are substantial increases from previous decades [10]. To counter these trends, the government is considering several options. One policy under consideration is mandatory front-of-pack (FOP) labeling on nutrition content (www.reach.gov.sg). FOP labeling has been established as an effective tool to improve diet quality [11,12] and is now mandated in several countries including Chile and Ecuador [13], while numerous others, including Australia and New Zealand, have introduced voluntary labelling to complement the Nutrition Information Panel (NIP) appearing on the back of many products. 

**Figure 1:**
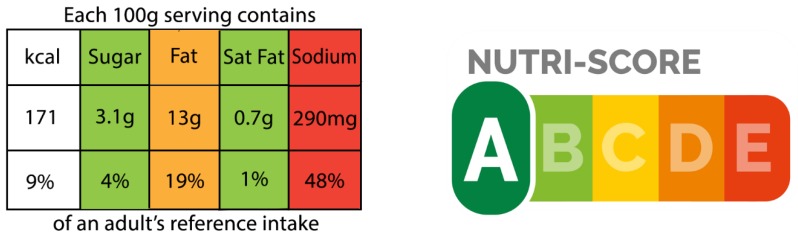
Front-of-pack labels under consideration in Singapore.

Two labels not currently used but under consideration in Singapore are the United Kingdom’s Multiple Traffic Light (MTL) label (Figure 1, left panel) and France’s Nutri-Score (NS) label (Figure 1, right panel). For each food/beverage, the MTL separately presents major nutrient information with individual color-coded ratings for each nutrient based on reference intakes and the guidelines set out by the European Food Information Council. Contrarily, the French NS label presents a single summary score representing the overall diet quality for each food/beverage on a five-point color-coded scale from green (best) to red (worst) using the British Food Standards Agency nutrient profiling system.

Both labels have been shown to be effective against no label control conditions or against other FOP labels [14]. However, only hypothetical head-to-head studies that do not involve actual purchases have been conducted [15,16]. These studies, based on hypothetical purchases, suggest that NS may outperform MTL in promoting overall diet quality, possibly because of the high cognitive load required to understand the MTL and because seeing multiple attributes, with some good and some bad, may create decisional conflicts [17]. NS resolves both of these concerns by providing a single summary measure of the diet quality of the food/beverage. Therefore, although we hypothesize that both the MTL and NS labels will improve overall diet quality, as measured via a modified version of the Alternative Healthy Eating Index-2010 (AHEI-2010) or the weighted (by servings) average Nutri-Score of the shopping basket (with A = 5 and E = 1), relative to a no-label Control, we expect the effect will be greater for the NS label. AHEI-2010 is a validated index of diet quality and higher scores have been strongly associated with lower risk of major chronic diseases [7]. However, for singular measures of diet quality, including total and per serving values of: sugar (g), energy (kcal), fat (g), saturated fat (g), sodium (mg), fiber (g) and protein (g), we hypothesize that MTL will outperform NS as those who care specifically about these measures will see the values directly on the MTL label along with the percentage of daily recommended intakes. 

We also hypothesize that being hungry or in an unhappy mood at the time of shopping moderates the effectiveness of the labels. This is possible as negative mood and hunger have been associated with greater impulsivity [18]. Thus, shoppers with these attributes may be more likely to ignore the labels altogether. We also test for moderating effects of education and income. Those with greater education may be more likely to pay attention to and use the labels [19,20] and because healthier foods tend to be more expensive [21], income may also moderate the effect of any FOP label. As healthier foods tend to be more expensive, we also test whether either label increases total expenditure per shopping trip ($) and calorie per dollar (kcal/dollar). We test these hypotheses using an experimental online grocery store in Singapore where food is purchased and delivered to participants’ homes. 

## 2. Materials and Methods 

### 2.1. Design and Participants

Participants were recruited via Facebook and Instagram advertisements from September to November 2018. Prospective participants were directed from digital posters to the study website and asked to complete an online screener to determine eligibility. Prospective participants were offered the chance to participate if they were residing in Singapore, 21 years of age or above, and registered RedMart (a large on-line retailer in Singapore) shoppers. Recruiting existing online grocery shoppers ensured that participants would be familiar and comfortable with online shopping. 

Those interested and eligible were asked to complete: 1) a registration form containing name, mobile phone number, and email address; 2) an online consent form; and 3) after obtaining consent, a baseline demographics questionnaire. The study purpose and research hypotheses were not revealed to participants until their participation concluded, as this could have influenced their purchasing behavior. All participants received a debriefing summary with full details of the study upon study completion. Upon completion of all forms, the website created the participant’s account and unique participant identification number (PID) for use throughout the study. Participants then received an automated email with their unique login details and were asked to logon to the NUSMart online grocery store to complete the first of three shopping tasks. 

NUSMart is an online experimental grocery store developed by the study team and used to run the present trial (https://nusmart.duke-nus.edu.sg/NM). At the time of the trial, NUSMart contained over 4,000 products commonly purchased in local supermarkets (food and beverages only) in Singapore. Food and beverage items are sorted into various categories, such as dairy products, carbonated soft drinks, fresh meats and seafood, and snacks. Participants are able to add and remove products to and from their online grocery cart and review their cumulative total cart cost. The online grocery store was designed to mirror actual online grocery stores available in Singapore such as Fairprice Online (https://www.fairprice.com.sg/) and RedMart (https://redmart.lazada.sg/#home) in look and feel. All products include pictures of the item, retail prices and product descriptions. Nutrition Information Panels and product information are available on click-through.

Over the course of three weeks, participants logged on to the NUSMart website once a week and were asked to purchase their weekly groceries with a minimum spend of $50 and maximum spend of $100. Each participant therefore shopped a total of three times during the study, including one shop in each of three shopping conditions. A minimum and maximum spend ensured that participants completed a typical weekly grocery order and that no outliers would skew the data.

Participants shopped with the knowledge that, for each shop, they had no more than a 1 in 3 chance of being required to purchase the chosen foods using their credit card. The requirement to purchase would only be revealed upon spinning a digital “Wheel of Purchase” after hitting the checkout button that allowed for recording their weekly shop. This design was chosen to increase the likelihood that the purchases were an accurate reflection of the participants’ actual shopping patterns, lending credibility of the results over alternative designs that rely only on hypothetical shops. The grocery orders that needed to be fulfilled were repurchased by the study team and delivered via RedMart.

Upon completion of each order, participants completed a short post-shop survey to establish their mood and how hungry they were at the time of placing their order. This information was analyzed to test if these variables moderated the influence of the labels. Participants that completed all study elements were compensated with a $75 Lazada electronic gift voucher (a popular eCommerce website in Singapore, https://www.lazada.sg). 

The study protocol was approved by the Institutional Review Board, National University of Singapore (S-18-189) and registered on Clinicaltrials.gov under the number NCT03761342. All investigations were conducted according to the principles expressed in the Declaration of Helsinki and all participants provided written (electronically online) informed consent before being enrolled in the study. 

### 2.2. Interventions and Outcomes

The study was a crossover trial where all participants were exposed once to three shopping conditions in random order. Participants were randomly assigned to one of six intervention sequences via random permuted blocks of size three with equal allocation for the six sequences (see Appendix A Table A8) by a computer program. Participants were blinded to intervention allocation, which was allocated via the NUSMart system. Allocation results were recorded within NUSMart and all investigators were blinded to group allocation. 

**Figure 2:**
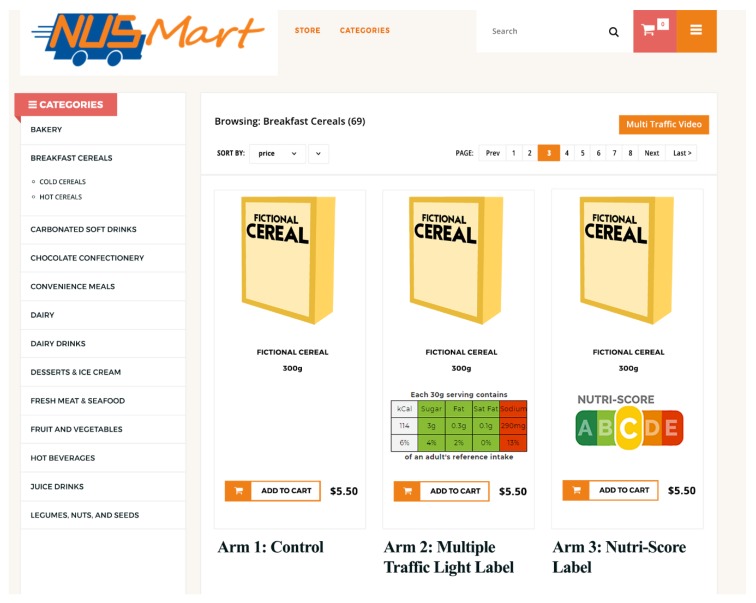
Example of the storefront with a sample of the Multiple Traffic Light and Nutri-Score labels on the same product as it appears in each condition.

Arm 1 was a Control condition that mirrors a traditional web-grocery store with back-of-pack NIPs, but with no FOP labels. Arm 2 (MTL condition): is similar to Arm 1, with Multiple Traffic Light labels displayed on the FOP of all products. MTL was applied based on the NFP without modification of the original algorithm [22]. Arm 3 (NS condition) is similar to Arm 2, with Nutri-Score labels instead of MTL labels displayed on the FOP of all products. For the 3343 foods in the store, Nutri-Score was applied without modification of the original algorithm [23]. 26% (876 products) received an A grade, 12% (409 products) received a B grade, 26% (880 products) received a C grade, 25% (849 products) received a D grade, and 9.8% (329 products) received an E grade. For the 832 beverages, we applied a modified score based on the Singapore Health Promotion Board’s (HPB) proprietary scoring system that maintains a greater focus on calories and sugar content and aims to provide a greater distribution of scores compared to using NS without modification. Using NS without modification, 87% of drinks would have been assigned a D or E grade. Under the modified scoring: 29% that contained no sugar were assigned an A grade, 11% received a B grade, 3% received a C grade, 3% received a D grade, and 54% received an E grade. Appendix A Table A9 shows the full list of beverages and their scores. Figure 2 shows an example of the NUSMart storefront with a sample of the MTL and NS labels as they appeared on the same fictional product in each condition. 

Prior to each shopping trip, a 60-second introductory video briefly explaining the MTL or NS labels was shown to participants in the corresponding condition. This sought to educate shoppers about how to read and understand each label, given that the local population has limited exposure to and education on the MTL and NS labeling schemes. The store was set up such that a participant could not shop until the videos were viewed.

All outcomes were calculated using the sales orders submitted by participants via the online system. The primary outcome is diet quality per shopping trip, as measured by a modified index of diet quality, the Alternative Healthy Eating Index (AHEI-2010). The AHEI-2010 is an updated measure of diet quality from the original Alternate Healthy Eating Index. It was constructed based on foods and nutrients predictive of chronic disease risk that incorporate current scientific evidence on diet and health; higher scores on the AHEI-2010 are strongly associated with a lower risk of major chronic diseases and cardiovascular mortality [7]. Eleven of 13 AHEI-2010 components, including vegetables, fruits, whole grains, sugar-sweetened beverages and fruit juice, nuts and legumes, red/processed meat, trans fat, long-chain fats and sodium are scored from 0 (worst) to 10 (best) were used for the present study. We dropped alcohol because it is not sold in our store and polyunsaturated fatty acids, as this information was not available. Therefore a maximum score of 90 (perfect diet quality) as compared to the usual maximum of 110 was employed. As we were calculating the scores on a weekly grocery purchase for the household, we divided the grocery purchase by the number of adult household members and by seven days to obtain the “per-day-per-person” consumption. Our secondary measure of diet quality, average Nutri-Score of the shopping basket, weighted by serving size, was calculated by applying A = 5 down to E = 1 for each food purchased. Other secondary outcomes included per serving and total values of calories, saturated fat, total fat, sodium, and sugar, and to quantify the effect of the labels, total spend and calories per dollar spent. 

### 2.3. Statistical Methods

A power calculation revealed that 140 participants were required to detect effect sizes of 0.30 or larger between arms. The calculation assumed a two-tailed test, a significance level of 0.05, power of 0.90, adjustment for 3 comparisons, and a cross-over design. 

Data is analyzed from an intention-to-treat approach that conforms to the Consolidated Standards of Reporting Trials (CONSORT) standards for reporting of randomized trials. The primary analysis employs the following first-differenced model:

(1) $$\Delta Modified\;AHEI_{is}=\alpha +\beta_{NS}NS+\epsilon_{is}.$$

The first difference model exploits the repeated observations of individuals by differencing away, and thus controlling for, time invariant heterogeneity (e.g., age, health consciousness etc.) between individuals. In this specification, the dependent variable is the difference in modified AHEI scores in each treatment condition and the Control condition. The constant term, α, measures the difference in the Modified AHEI score for the MTL condition relative to the control shop. NS is a dummy variable that is set to one for shops where NS labels appeared on all products. $\epsilon_{is}$ is the error term. The subscripts are for each individual, i, and each shop, s. Each participant generates two observations (NS vs control and MTL vs control). This model is then estimated via ordinary least squares (OLS) with errors clustered at the individual level, so as to account for correlation between repeated shops for the same individuals. Secondary outcomes are analyzed using the same model. With the exception of the modified AHEI-2010, which is a composite measure of the entire basket, we also run the above models separately for foods and beverages. AHEI-2010 should not be run for food and beverages separately since the index scores are dependent on dietary components that span all major food and drink groups, and are not scored in isolation.

In line with our hypotheses, we conduct the following tests:

1. α>0, Testing for whether the outcome is significantly greater in MTL than control;
2. $\alpha +\beta_{NS}>0$, Testing for whether the outcome is significantly greater in NS than control;
3. $\beta_{NS}>0$, Testing for whether the difference in outcome is greater for NS than for MTL.

To test the moderating effects of mood, hunger, income and level of education, we interacted moderators with the treatment dummy, resulting in the below model:

(2) $$\Delta Modified\;AHEI_{is}=\alpha +\beta_{NS}NS+\beta_{M}Moderator+\beta_{int}Moderator*NS+\epsilon_{is},$$

where Moderator values greater than or equal to the median values for hunger and mood are considered hungry and happy respectively. $\beta_{M}>0$ tests for whether the moderators (i.e., hungry, happy, high income, high level of education) differentially influence the relationship between MTL and AHEI scores. $\beta_{M}+\beta_{int}>0$ tests this relationship for NS. Each moderator regression was run separately. All statistical analyses were performed using STATA version 15.

## 3. Results

### 3.1. Sample

**Figure 3:**
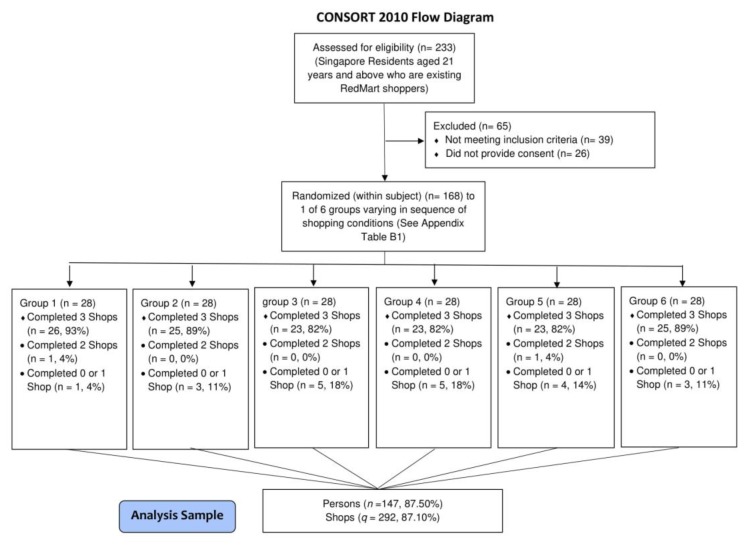
CONSORT flow diagram for participant recruitment and randomization.

Participant flow and corresponding sample sizes are presented in Figure 3. 168 participants were recruited to participate in the study. 145 completed all three shops. 14 did not place any order, 7 placed one and 2 placed two orders. Using the first difference model, we could analyze those with at least two purchases but not those with only one, resulting in an analysis sample of 147 participants. Table 1 presents the characteristics of this sample. The sample was largely of Chinese ethnicity (93.51%) and the mean age was 34.40 years (standard deviation (SD) = 6.88). The average body mass index (BMI) was 23.31 kg/m^2^ (SD = 4.07). The majority (68.83%) were female. The characteristics of participants who dropped out did not differ from the analysis sample. The completed CONSORT checklist can be accessed as Supplementary Materials.

Table 2 presents the unadjusted values of the primary and secondary outcomes for the Control. Regression output testing for differences between conditions is reported in Table 3.

The mean modified AHEI-2010 score was 41.81 in Control. Consistent with our hypothesis, both labels showed statistically significant increases in modified AHEI-2010 scores (Table 3). The estimated increase was 1.09 in NS (*p* = 0.04) and 1.16 in MTL (*p* = 0.04) compared to Control. Contrary to expectations, the effect was not significantly different between labels. The mean Nutri-Score was 3.41. There was an increase in Nutri-Score by 0.33 points (*p* < 0.01) in NS compared to Control and, consistent with our hypothesis, by 0.31 points (*p* < 0.01) compared to the estimated effect of MTL. The difference in average Nutri-Score of 0.02 between MTL and control was not statistically significant (*p* = 0.06). 

There were no significant moderating effects of mood, hunger, education, or income on the modified AHEI-2010 or average Nutri-Scores, suggesting that these factors did not differentially influence the effect of labelling on diet quality for on-line shopping. The results can be found in Table A7 in the Appendix A.

### 3.2. Estimated Treatment Effect on Secondary Outcomes

In MTL, consistent with our hypothesis, calories decreased by 19.75 kcal/serving (*p* = 0.01), fat decreased by 1.03g/serving (*p* = 0.03) and protein decreased by 0.83g/serving (*p* = 0.01) compared to Control. In NS relative to Control, total saturated fats per order decreased by an estimated 29.29 g (*p* = 0.01). No other differences were statistically significant. These results can be found in Table A1 and Table A2 of the Appendix A.

### 3.3. Treatment Effect on Foods

For the analyses on food items only, there was a significant effect of the NS condition on average Nutri-Score (0.21, *p* = 0.02) and total saturated fats (–29.79g, *p* = 0.01) relative to control. MTL led to a significant decrease of calories per serving by 20.56kcal/serving (*p* = 0.02) and fats per serving by 1.02g/serving (*p* = 0.03) relative to control. No other differences were statistically significant. Results of the food-only analyses can be found in Table A3 and Table A4 in the Appendix A.

### 3.4. Treatment Effect on Beverages

For the analyses on beverages only, NS improves average Nutri-Score by 0.72 (*p* < 0.01), with NS having a significantly higher effect than MTL by 0.52 (*p* = 0.01). Similar to the total basket, relative to control, MTL reduced calories, fats and protein per serving by -15.48 kcal (*p* = 0.01), −0.55g (*p* = 0.03) and −0.76 g (p = 0.01), respectively. MTL also decreased total sugar purchased by −66.83 g (*p* = 0.03). Results of the drinks-only analyses can be found in Table A5 and Table A6 in the Appendix A.

## 4. Discussion

Front-of-pack labeling has been identified by the Singapore government as one of four promising strategies to tackle nutrition-related diseases. Our results show that both the Multiple Traffic Lights and Nutri-Score labeling scheme employed led to statistically significant improvements in diet quality relative to no-labelling based on mean modified AHEI-2010 scores and average Nutri-Score. In the Singapore Chinese population, a 10-point higher AHEI-2010 was associated with a 21% lower risk of coronary artery disease [24]. Thus, assuming a linear relationship between improvements in AHEI and reductions in coronary artery disease, the observed 1.16 point improvement, were it to be sustained on a population level, could be associated with as much as a 2.4% decrease in coronary artery disease, which is a relative large effect for a low-cost intervention. Testing the effects of the label on risk factors for non-communicable diseases should be an area of future research. Neither label was superior to each other in terms of modified AHEI-2010 scores, but NS performed better than MTL in terms of average Nutri-Score. This should not be surprising given that the average Nutri-Score most closely tracks what consumers saw on the NS label. 

These results provide support for implementation of either label if the goal is to improve overall diet quality as assessed via modified AHEI-2010 and average Nutri-Score. However, if the goal is to reduce intake of calories or improve intake of a specific nutrient, MTL may be preferred. Unlike NS, MTL showed statistically significant reductions in calories and fats per serving purchased relative to control in the full basket, and reductions in total sugar purchased for beverages. This result should not be surprising if we believe calories, sugar and fat are what consumers are most concerned about. MTL allows these nutrients to be seen directly on the label along with per serving values as a percentage of daily recommended values, whereas NS only shows a summary measure that masks potential benefits (i.e., decreases) in these individual nutrient domains. As a result, if the goal is to decrease calorie and sugar intake or rates of obesity, as opposed to improve overall diet quality, MTL may be preferred. Our results did not show MTL to be statistically different in these specific domains relative to NS, but this may be due to a lack of statistical power. 

Moderator analyses revealed no differential effects by mood, hunger, education or income on measures of diet quality. Although other studies have found mood and hunger to influence impulsivity and attentiveness when shopping for food [18,25,26,27,28], and these may influence the effects of FOP labels, this effect may be attenuated for on-line shopping where purchases and consumption and more disconnected. The lack of differential effects by education and income is encouraging as it suggests all shoppers may equally benefit from the labels. However, all moderating results should be viewed with caution given that the study may have been underpowered to identify these differences. 

This study had several strengths including its within-person randomized controlled trial design, a fully functional online grocery store with thousands of products to choose from, and actual delivery of orders for a subset of shops so that consumer shopping behavior was more likely to mimic an actual purchase. However, the study also has several potential limitations. Shopping was limited to one shop per condition and results may differ with repeated shops. The relative effectiveness of each label may also differ if the underlying algorithms used to define the labels were modified or if alternative summary measures of diet quality were used. AHEI is an accepted measure of diet quality at the individual level but is less frequently used to assess the quality of shopping baskets. Average Nutri-Score is less common but, as we show in Appendix A Table A10, baskets that score better in this value also score better in AHEI and in each measure of diet quality listed on the NFP. However, as it is (by design) most strongly correlated with the NS label, it is biased in favor of this label. Future studies should consider alternative measures of overall diet quality. On-line shopping may also differ from in-store shopping, making it difficult to generalize the findings of this study to brick-and-mortar stores. Our sample is also not representative of the broader population. It includes a greater percentage of females, Chinese, and those with university education. Future studies should test these labels over repeated purchases and in different shopping venues, including on-line and in-person grocery and convenience stores, and with a broader subset of the population to explore whether results are sustained and generalizable and to identify which population subsets (e.g., dieters, more nutrition knowledgeable) are most likely to benefit from each type of label. Furthermore, showing participants the 60-second introductory videos may have primed them to shop for healthier products. However, given their lack of exposure to these labels we found it necessary to educate them on how to use the new labels as would occur with any effort to implement such labels in Singapore. As a final limitation, it is worth noting that our trial focused on the effects of FOP labelling on consumer choices, but effective labels could also encourage suppliers to reformulate (a positive outcome) and/or change prices in ways that may undermine some of the positive effects of the label. Real world studies are needed to explore these effects. 

## 5. Conclusions

Both the MTL and Nutri-Score front-of-pack labels improved dietary quality according to the modified AHEI-2010, providing support for implementation of either label if the goal is to improve overall diet quality. NS outperforms MTL and no FOP labels in average Nutri-Score but, unlike MTL, does not reduce calories, suggesting that MTL may be preferred to NS if the goal is to reduce caloric intake and obesity rates. 

## Figures and Tables

**Table 1:** Descriptive statistics.

Variable	Analysis Sample (*n* = 147)	Drop-Out Sample (*n* = 21)
	Mean/% (S.D.)	Mean/% (S.D.)
Age (years)	34.69 (6.83)	33.17 (8.12)
Body mass index (BMI) (kg/m^2^)	23.31 (4.07)	24.06 (4.96)
Female (%)	68.71 (5.62)	83.33 (4.52)
Ethnicity (% Chinese)	93.20 (3.05)	100 (0)
University education and above (%)	67.01 (5.70)	66.67 (5.72)
Household income $10,000/month and above (%)	32.65 (5.69)	12.50 (4.01)

**Table 2:** Dietary characteristics of food purchases in the Control condition (*N* = 147).

Outcome	Unadjusted Mean	95% CI
Modified Alternative Healthy Eating Index (AHEI-2010) Score	41.81	(40.71, 42.92)
Average Nutri-Score	3.41	(3.33. 3.50)
Total energy (kcals) (in 1000s)	9.72	(8.40, 11.04)
Total sugar (g) (in 1000s)	2.68	(2.25, 3.10)
Total fat (g) (in 1000s)	4.41	(3.38, 5.43)
Total saturated fat (g) (in 1000s)	1.57	(1.02, 2.12)
Total sodium (mg) (in 1000s)	108.74	(80.94, 136.53)
Total fiber (g) (in 1000s)	0.92	(0.73, 1.11)
Total protein (g) (in 1000s)	3.16	(2.59, 3.73)
Mean calories per serving (kcal/serving)	127.74	(117.60, 137.87)
Mean sugar per serving (g/serving)	4.57	(3.80, 5.35)
Mean fat per serving (g/serving)	5.21	(4.59, 5.83)
Mean saturated fat per serving (g/serving)	1.83	(1.58, 2.08)
Mean sodium per serving (mg/serving)	144.49	(116.95, 172.03)
Mean fiber per serving (g/serving)	1.19	(1.01, 1.37)
Mean protein per serving (g/serving)	4.66	(4.03, 5.28)
Total spend ($)	53.86	(52.41, 55.31)
Calorie per dollar (kcal/$)	249.63	(231.20, 268.05)

**Table 3:** Estimated effects of Multiple Traffic Lights (MTL) and Nutri-Score labels (*n* = 147, *N* = 292).

Outcome	AHEI-2010	Average Nutri-Score
α (MTL vs Control)	1.16 *	0.02
(s.e.)	(0.53)	(0.08)
$\beta_{NS}\;$ (incremental effect of NS over MTL)	−0.07	0.31 **
(s.e.)	(0.58)	(0.09)
($\alpha +\;\beta_{A})$ (NS vs Control)	1.09*	0.33 **
(s.e.)	(0.53)	(0.09)

* *p* < 0.05, ** *p* < 0.01.

## Supplementary Materials

The following are available online at https://www.mdpi.com/2072-6643/11/9/2236/s1: Table S1: CONSORT 2010 checklist of information to include when reporting a randomised trial.

## Appendix A

**Table A1:** Regression with dietary quality outcomes and per serving outcomes (*N*, the number of differences = 292, *n, the number of individuals* =147).

Outcome	Mean per Serving Values								
	AHEI-2010	Avg NS	Energy (kcal)	Sugar (g)	Fats (g)	Sat. Fats (g)	Sodium (mg)	Fiber (g)	Protein (g)
$\beta_{A}$ NS (incremental effect of NS over MTL)	−0.07	0.31 **	11.43	−0.13	−0.64	0.08	17.07	0.03	0.66
(s.e)	(0.57)	(0.09)	(7.55)	(0.80)	(0.52)	(0.16)	(19.52)	(0.09)	(0.34)
α (MTL vs. Control)	1.16*	0.02	19.75 *	−0.59	−1.03 *	−0.29	−30.46	−0.02	−0.83 *
(s.e.)	(0.53)	(0.08)	(7.25)	(0.15)	(0.48)	(0.17)	(17.73)	(0.07)	(0.32)
$\beta_{A}+\alpha$ (Effect of NS)	1.09*	0.33 **	−8.32	−0.72	−0.39	−0.22	−13.39	0.01	−0.17
(s.e)	(0.53)	(0.09)	(5.20)	(0.48)	(0.30)	(0.17)	(19.15)	(0.09)	(0.29)

* *p* < 0.05, ** *p* < 0.01

**Table A2:** Regression with total value outcomes (*N* = 292, *n* =147).

Outcome	Total Values								
	Energy (kcal)	Sugar (g)	Fats (g)	Sat. Fats (g)	Sodium (mg)	Fiber (g)	Protein (g)	Total expenditure ($)	kcal/$
$\beta_{A}$ NS (incremental effect of NS over MTL)	199.15	3.35	−33.83	−9.85	−1729.90	6.90	8.60	−0.51	5.28
(s.e)	(758.97)	(23.53)	(57.82)	(10.68)	(1092.24)	(7.25)	(15.21)	(0.748)	(13.56)
α (MTL vs. Control)	−951.39	−52.02	36.89	−19.44	−266.53	0.80	−67.97	0.26	−8.08
(s.e.)	(1039.70)	(30.61)	(44.11)	(13.58)	(806.73)	(6.62)	(43.67)	(0.723)	(13.02)
$\beta_{A}+\;\alpha$ (Effect of NS)	−752.2	−48.67	3.07	−29.29 **	−1996	7.70	−59.38	−0.25	−2.81
(s.e)	(104.02)	(28.09)	(51.35)	(10.96)	(1032.00)	(6.87)	(43.88)	(0.853)	(12.79)

* *p* < 0.05, ** *p* < 0.01.

**Table A3:** Regression for foods only dietary quality and per serving values as outcomes (*N* = 287, *n* = 146).

Outcome	Mean per Serving Values							
	Avg NS	Energy (kcal)	Sugar (g)	Fats (g)	Sat. Fats (g)	Sodium (mg)	Fiber (g)	Protein (g)
$\beta_{A}$ NS (incremental effect of NS over MTL)	0.31 **	10.34	−0.90	0.39	−0.09	5.83	−0.01	0.52
(s.e)	(0.09)	(8.49)	(0.73)	(0.60)	(0.21)	(24.58)	(0.11)	(0.39)
α (MTL vs. Control)	−0.10	−20.56 *	0.38	−1.02 *	−0.24	−38.58	−0.04	−1.16
(s.e.)	(0.09)	(8.56)	(0.69)	(0.53)	(0.22)	(24.29)	(0.10)	(0.51)
$\beta_{A}+\;\alpha$ (Effect of NS)	0.21 *	−10.22	−0.52	−0.64	−0.33	−32.75	−0.05	−0.64
(s.e)	(0.09)	(6.30)	(0.40)	(0.47)	(0.19)	(24.13)	(0.10)	(0.53)

* *p* < 0.05, ** *p* < 0.01.

**Table A4:** Regression for foods only with total values and cost as outcome (*N* = 287, *n* = 146).

Outcome	Total Values								
	Energy (kcal)	Sugar (g)	Fats (g)	Sat. Fats (g)	Sodium (mg)	Fiber (g)	Protein (g)	Total expenditure ($)	Kcal/$
$\beta_{A}$ NS (incremental effect of NS over MTL)	303.78	2.50	−32.55	−10.97	−1705.24	5.18	16.02	−0.23	32.32
(s.e)	(776.24)	(17.49)	(58.73)	(10.65)	(1103.66)	(6.90)	(15.97)	(0.95)	(22.82)
α (MTL vs. Control)	−799.41	−9.42	36.48	−18.83	−204.48	5.24	−67.58	1.13	−29.36
(s.e.)	(1103.95)	(24.05)	(45.62)	(13.41)	(843.21)	(6.11)	(43.34)	(1.06)	(20.17)
$\beta_{A}+\;\alpha$ (Effect of NS)	−6.92	−495.6	3.94	−29.79 **	−1910	10.42	42.94	0.90	2.96
(s.e)	(21.13)	(1087.00)	(52.78)	(10.91)	(1045.00)	(6.38)	(0.23)	(0.98)	(19.22)

* *p* < 0.05, ** *p* < 0.01.

**Table A5:** Regression for beverages only with dietary quality and per serving values outcomes (*N* = 190, *n* = 111).

Outcome	Mean per Serving Values							
	Avg NS	Energy (kcal)	Sugar (g)	Fats (g)	Sat. Fats (g)	Sodium (mg)	Fiber (g)	Protein (g)
$\beta_{A}$ NS (incremental effect of NS over MTL)	0.52 **	9.34	0.12	0.87 *	0.70 **	10.62 *	0.13	0.48
(s.e)	(0.196)	(6.99)	(0.98)	(0.32)	(0.21)	(4.58)	(0.12)	(0.32)
α (MTL vs. Control)	0.20	−15.48 **	−1.83	−0.55 **	−0.18	−4.30	−0.06	−0.76 **
(s.e.)	(0.168)	(6.20)	(0.95)	(0.25)	(0.14)	(4.46)	(0.08)	(0.27)
$\beta_{A}+\;\alpha$ (Effect of NS)	0.72 **	−6.14	−1.71	0.31	0.52	6.31	0.07	−0.28
(s.e)	(0.18)	(6.76)	(0.93)	(0.34)	(0.20)	(4.64)	(0.12)	(0.36)

* *p* < 0.05, ** *p* < 0.01.

**Table A6:** Regression for beverages only with total values and cost as outcome (*N* = 190, *n* = 111).

Outcome	Total Values								
	Energy (kcal)	Sugar (g)	Fats (g)	Sat. Fats (g)	Sodium (mg)	Fiber (g)	Protein (g)	Total expenditure ($)	Calorie/$
$\beta_{A}$ NS (incremental effect of NS over MTL)	186.97	28.09	5.03	6.04	−24.55	1.84	−2.51	0.78	20.93
(s.e)	(199.59)	(23.60)	(9.48)	(5.53)	(151.30)	(2.64)	(8.35)	(1.06)	(13.00)
α (MTL vs. Control)	−374.78	−66.83 *	−5.16	−3.81	−194.26	−4.82	−9.60	−1.48	−1.52
(s.e.)	(247.29)	(0.03)	(7.98)	(4.03)	(151.10)	(3.50)	(9.11)	(1.08)	(15.97)
$\beta_{A}+\;\alpha$ (Effect of NS)	−187.8	−38.74	−0.14	2.23	−218.8	−2.98	−12.12	−0.70	−10.60
(s.e)	(213.80)	(27.03)	(7.83)	(4.67)	(139.20)	(3.56)	(8.15)	(1.01)	(12.71)

* *p* < 0.05, ** *p* < 0.01.

**Table A7:** Regression with moderator analyses (*N* = 287, *n* = 146).

Outcome	Mood		Hunger		Income		Education	
	AHEI-2010	Avg NS	AHEI-2010	Avg NS	AHEI-2010	Avg NS	AHEI-2010	Avg NS
$\beta_{A}$ (NS)	−0.42	0.27	−1.58	0.21	0.31	0.36 **	−0.60	0.23
(s.e)	(0.83)	(0.14)	(0.89)	(0.15)	(0.68)	(0.12)	(1.35)	(0.20)
$\beta_{2}$ (Moderator)	−1.85	−0.34 *	1.81	0.13	1.79	−0.14	−0.82	−0.28
(s.e)	(1.06)	(0.17)	(1.06)	(0.17)	(1.19)	(0.16)	(1.32)	(0.23)
$\beta_{3}$ (Moderator × NS)	0.75	0.10	−3.19 *	0.19	−1.15	−0.16	0.62	0.10
(s.e)	(1.35)	(0.22)	(1.28)	(0.22)	(1.23)	(0.18)	(1.48)	(0.22)
$\beta_{2}+\;\beta_{3}$	−1.10	−0.24	−1.38	0.17	0.64	0.18	−0.19	−0.18
(s.e)	(1.07)	(0.16)	(1.07)	(0.06)	(1.13)	(0.10)	(1.15)	(0.23)
α (Constant)	2.08 **	0.19	0.25	−0.04	0.58	0.07	1.86	0.26
(s.e.)	(0.77)	(0.11)	(0.78)	(0.13)	(0.62)	(0.11)	(1.18)	(0.21)

* *p* < 0.05, ** *p* < 0.01.

**Table A8:** Six intervention sequences via random permuted blocks of size three with equal allocation.

Group Number	First shop	Second Shop	Third Shop
1	Baseline	MTL	NS
2	Baseline	NS	MTL
3	MTL	Baseline	NS
4	MTL	NS	Baseline
5	NS	Baseline	MTL
6	NS	MTL	Baseline

**Table A9:** Product names and Nutri-Score grades of all beverages sold on NUSMart.

Product Name	Assigned Grade
Gold Kili 2-in-1 Kopi Premium Coffee Mixture Bag with Sugar Added	B
Nutrisoy Plain Soymilk	B
Magnolia Fresh Milk	B
Nutrisoy Fresh Soyamilk—Reduced Sugar	B
Polleney Pure Soybean Powder No Added Sugar	B
Dilmah Premium Quality 100% Pure Ceylon Tea	A
Marigold 0% Fat Yoghurt Drink—Strawberry	B
Indocafe White Coffee	E
Gold Kili Instant 2-in-1 Espressccino	E
Living Planet Full Cream Organic Dairy Milk	E
Mirinda Orange	E
Ceres Secrets of the Valley 100% Juice	E
Red Bull Classic Energy Drink 25% Less Sugar	B
MILO UHT Chocolate Malt Packet Drink	E
Meiji Lowfat Milk	E
Meiji Lowfat Milk	E
Indocafe 3 in 1 Coffeemix	E
Regilait Milk Powder	E
Lipton Tea Granules	A
Magnolia Low Fat Hi Cal Milk	B
Nature 2000 Green Rooibos Tea	A
Marigold Peel Fresh No Sugar Added Juice Drink - Orange	B
Pokka Jasmine Green Tea	A
Sunkist Orange Juice—No Added Sugar	C
Segafredo Espresso Casa Coffee Beans	A
Boncafe All Day Coffee Beans	A
Pocari Sweat Ion Supply Drink	E
Marigold Peel Fresh No Sugar Added Juice Drink - Orange	B
Vitasoy Original Soy Drink	B
Sunkist Premium 100% Orange Juice	C
Farmhouse Low Fat Milk	E
Greenfields Skim Milk	E
Marigold Low Fat UHT Milk	B
Coca-Cola Zero Sugar	A
Milo Australian Recipe Malt Drink	E
Greenfields Low Fat Milk	E
Gold Kili Instant 3-in-1 Milk Tea	E
Farmhouse Fresh UHT Milk	A
Pocari Sweat ION Supply Drink	E
Marigold 100% Apple Juice	D
Cowhead UHT Pure Lite Milk	E
Cowhead UHT Pure Milk	E
7-UP Revive Original	E
Robert Timms Premium Rich and Smooth Freeze Dried Coffee	A
Nescafe Milk Coffee Original Can	E
Robinsons Orange and Mango No Added Sugar	E
Chen Jiah Juang Mulberry Vinegar Drink	E
Meiji Chocolate Flavour Milk	E
Orangina Sparkling Citrus Beverage	E
Yeo’s Bandung Drink	B
F&N Fruit Tree Fresh Carrot with Wolfberry—No Added Sugar	C
F&N Fruit Tree Fresh Orange - No Added Sugar	C
Milo UHT Malt Drink	E
Marigold Peel Fresh No Sugar Added Juice Drink—Powerberries	C
Lipton Yellow Label Tea (Enveloped Bag)	A
H-TWO-O Sparkling Isotonic Drink	D
Marigold Kiwi Juice Drink	C
Pokka Pomegranate Juice	E
Cadbury 3in1 Hot Chocolate Drink	E
Pokka Sparklin’ Fuji Apple Sparkling Fruit Drink	E
Super Regular 3-in-1 CoffeeMix	E
Tipco 100% Beetroot Formula Mixed Vegetable and Fruit Juice	E
Marigold Peel Fresh No Sugar Added Juice Drink—Powerveggies and Mixed Fruits	E
Charlie’s Fresh Squeezed Orange Juice	E
Original Juice Co Pineapple Juice (Not From Concentrate)	E
Pureharvest Organic Aussie Dream Rice Milk	E
Marigold Plant Sterols Plus HL Milk	A
Boncafe Espresso Gourmet Ground Coffee	A
Schweppes Lemon Lime and Bitters Pet Bottle	E
Meiji Fresh Milk	A
Society Tea	A
Meiji Lowfat Chocolate Milk	E
Swiss Miss Marshmallow 3 in 1 Hot Chocolate Drink Sachets	E
7-Up	E
Yeo’s Chrysanthemum Light Tea	B
Boncafe Colombiana Instant Coffee	E
Bushells Classic Gourmet Instant Coffee	E
Super Rich 3-in-1 CoffeeMix	E
Old Town 3-in-1 White Milk Tea	E
Hsiang Yuan Instant Chrysanthemum Drink with Goji	B
Basilur Tea Tea Book Volume I Tea	A
Marigold Full Cream UHT Milk	B
Aquarius Isotonic Drink	B
Pukka Organic Three Mint Tea	A
Marigold Peel Fresh No Sugar Added Juice Drink - Cloudy Apple	C
Taylors of Harrogate Organic Chamomile Tea 20’S	A
Brooke Bond 3 Roses Tea Mindsharp	A
Basilur Tea Tea Book Volume IV English Tea	A
F&N Magnolia Higher Calcium Low Fat Milk	A
Basilur Tea English Afternoon Loose Leaf Tea	A
100 Plus Isotonic Drink	B
Robert Timms Premium Full-Bodied Granulated Coffee	A
Meiji Coffee Flavour Milk	E
Basilur Tea Darjeeling Loose Leaf Tea	A
Lipton Packet Tea	A
Super Low Fat Less Sugar 3-in-1 Instant Coffee	E
Basilur Tea Bouquet Green Freshness Green Tea	A
Magnolia Mixed Berry with Nata De Coco Yoghurt Drink	E
Basilur Tea Moroccan Mint Loose Leaf Green Tea	A
Emmi Swiss Yogurt Drink Blueberry	E
F&N Magnolia Chocolate Flavoured Milk	A
Super 2-in-1 Charcoal Roasted White Coffee	E
Gold Kili Instant Ginger Drink	E
Nestle Nespray Fortified Full Cream Milk Powder	E
NESCAFE Gold Blend Instant Soluble Coffee 50G	E
Greenfields Chocomalt Milk	E
Heaven and Earth Ayataka No Sugar Japanese Green Tea	A
Pitti Caffe Pittissima Cremoso	A
Vitasoy Melon Flavored Soy Drink	B
Ceres Pineapple 100% Juice	E
Jia Jia Heritage Herbal Tea	A
Yeo’s Grass Jelly Drink	E
Coca-Cola Diet Coke Cherry	A
Schweppes Slimline Indian Tonic 12 Per Pack	A
Minute Maid Pulpy Tropical	E
Nutrisoy Almond Soymilk	E
Horlicks Nutritious Malted Drink - Refill	E
Super Cereal Oat 4-in-1 Cereal Drink	E
Basilur Tea English Breakfast Loose Leaf Tea	A
Swiss Miss Milk Chocolate 3 in 1 Hot Chocolate Drink Sachets	E
Robert Timms Gold Colombia Style Coffee Bags	A
Ovaltine	A
Pokka Honey Lemon	D
Gryphon Hanami Tea	A
Teisseire Grenadine No Added Sugar Syrup with Stevia	E
Nestle Everyday Instant Filled Milk Drink Powder	E
Cowhead UHT Chocolate Milk	C
Dutch Mill Yoghurt Drink with Mixed Fruits Juice	E
Dutch Mill Yoghurt Drink with Orange Juice	E
Clipper Organic Infusion Peppermint Tea	A
F&N Fruit Tree Fresh Cranberry Pomegranate - No Added Sugar	C
H-TWO-O Sparkling Isotonic Drink	D
Horlicks Malt Chocolate 3-in-1	E
Lipton Peach Mango Pyramid Tea	B
Orangina Sparkling Citrus Beverage	E
F&N NutriSoy Soya Milk with Calcium and Reduced Sugar	A
Teisseire Raspberry and Cranberry No Added Sugar Syrup with Stevia	E
Schweppes Dry Ginger Ale	E
Vitagen Collagen Less Sugar Cultured Milk—Assorted	E
Vitagen Less Sugar Cultured Milk—Assorted	E
Gingen Ginger with Brown Sugar Natural Beverage	E
Dutch Mill Yoghurt Drink with Blueberry Juice	E
Orangina Sparkling Citrus Beverage	E
Dilmah Pure Peppermint Leaves	A
Pureharvest Organic Aussie Dream Enriched with Calcium Rice Milk	E
Ceres 100% Full Moon Harvest Juice	E
Super 3-in-1 Charcoal Roasted White Coffee with Brown Sugar	E
The Berry Company Blueberry	E
Daisy Low Fat High Calcium Plain Milk	B
Marigold 100% Fresh Milk—Plain	D
Ceres Orange 100% Juice	E
Lipton Forest Fruit Pyramid Tea	A
Origina Juice- Apple Flavour	E
Lipton Citrus Pyramid Tea	A
Coco Life Coconut Water	B
Dutch Lady UHT Strawberry Milk	E
Monin Vanilla Syrup	A
Monin Hazelnut Syrup	A
Taylors of Harrogate English Breakfast Tea Leaf Caddy	A
Tata Gold Tea	A
Pureharvest Organic Oat Milk Non-Dairy	E
Dutch Lady UHT Low Fat Milk	A
OSK 100% Japanese Green Tea	A
Basilur Tea Oriental Masala Chai	A
Clipper Fairtrade Citrus Green Tea with Echinacea	A
Sunkist 100% Pure Orange and Pink Grapefruit Juice—No Added Sugar or Flavour	E
Sunkist 100% Pure Orange Juice with lots of Pulp - No Added Sugar or Flavour	E
Sunkist 100% Pure Orange Juice with Pulp—No Added Sugar or Flavour	E
Ribena Blackcurrant and Strawberry Juice Cordial	D
Ribena Blackcurrant Fruit Drink	D
Ribena Blackcurrant and Strawberry Fruit Drink	D
Dutch Lady Full Cream Milk	A
Pukka Organic Three Ginger Tea	A
Les Vergers Du Mekong Coconut Water and Lime Fruit Juice	A
Greenfields Fresh Low Fat Milk	E
Unisoy Instant Soya Milk Powder	A
Unisoy Instant No Cane Sugar Added Soya Milk Powder	A
Oishi Green Tea Genmai	A
Ceres Guava 100% Juice	E
Pokka No Sugar Jasmine Green Tea	A
Gingen Ginger with Honey Natural Beverage	E
Allswell Golden Pear with Aloe Vera	D
Sunquick Lemon Squash Concentrate	A
Horlicks 3in1 Instant Original Nutritious Malted Drink	E
Nescafe Dolce Gusto Chococino	E
Gryphon Chamomile Dream Tea	A
Nutrisoy Omega Soymilk—Less Sugar	E
Nutrisoy Omega Soymilk—No Added Sugar	E
Farmhouse Low Fat Milk	E
Farmhouse Fresh Milk	E
Devondale UHT Full Cream Milk	E
Devondale UHT Skim Milk	E
F&N Magnolia Super Slim Low Fat Milk	A
Lipton Signature Classic Milk Tea Latte	E
Nestle Bliss Low Fat Mango and Peach Yoghurt Drink	E
Nestle Bliss Low Fat Tropical Fruit Yoghurt Drink	E
Nestle Bliss Low Fat Mixed Berries Yoghurt Drink	E
Super Cereal Original 3-in-1 Cereal Drink	E
Gold Kili Kopi-O Kosong Premium Coffee Mixture Bag	B
Nestum 3 in 1 Original Cereal Drink with Oats	E
NESCAFE Gold Blend Instant Soluble Coffee 200 g	E
Old Town 3-In-1 Hazelnut White Coffee Mix	E
Old Town 2-In-1 Coffee and Creamer White Coffee Mix	E
Gryphon Lemon Ginger Mint Tea	A
Gryphon British Breakfast Tea	A
Gryphon White Gingerlily Tea	A
Gryphon Earl Grey Lavender Tea	A
Cowhead Milk Powder	A
F&N Tonic Water	E
Oishi Green Tea Original	A
Oishi Green Tea Genmai	A
Greenfields Fresh Milk	B
Pauls Fresh Milk	E
NESCAFE CLASSIC Jar Instant Soluble Coffee 50 g	A
Owl Teh Tarik Instant Foamy Tea	E
Just Juice Orange Juice	E
Just Juice Pineapple Juice	E
Nescafe Menu Brown Sugar Flavour Ipoh White Coffee	E
Nescafe Menu Hazelnut Flavour Ipoh White Coffee	E
Heaven and Earth Ice Passionfruit Tea	E
Heaven and Earth Ice Lemon Tea	E
Charlie’s Fresh Squeezed Orange Juice	E
Charlie’s Fruit Fix Acai and Berry	E
Charlie’s Fruit Fix Spirulina	E
Boncafe All Day Gourmet Ground Coffee	A
Boncafe Morning Gourmet Ground Coffee	A
Ribena Cheerpack Blackcurrant Fruit Drink	D
Original Juice Co Orange Juice Squeezed (Not from Concentrate)	E
Orangina Sparkling Citrus Beverage	E
NESCAFE Blend 37 Instant Soluble Coffee 100 g	A
MILO Can Calcium Plus	B
Nescafe Collection Espresso	A
Vitasoy Original Soy Drink	B
Marigold 100% Carrot Mixed Fruits Juice	D
Marigold 100% Orange Juice	C
Marigold 100% Apple Cranberry Juice	D
Tan Ngan Lo Herbal Tea	D
Basilur Tea Four Seasons Winter Tea	A
Nescafe Gold Decaffeinated Instant Coffee	A
Cowhead UHT Chocolate Milk	C
Clipper Organic Infusion Chamomile Tea	A
Heaven and Earth Mango Tea with Chamomile	C
Red Bull Energy Drink	B
Charlie’s Lemonade Quencher	E
Charlie’s Mango and Orange Quencher	E
CocoMax 100% Coconut Water	B
Ceres Red Grape 100% Juice	E
Cowhead UHT Lactose Free Milk	E
Ceres Whispers of Summer 100% Juice	E
Dilmah Earl Grey Tea	A
The Berry Company Acai Berry	E
Cowhead UHT Slim Milk	E
Wong Coco All Natural Coconut Juice with Pulp	E
NESCAFE Milk Coffee Mocha Can	E
Taylors of Harrogate Yorkshire Tea 80 Tea Bags	A
Magnolia Oats Plus Low Fat Hi Cal Milk	A
Magnolia Omega Plus Low Fat Hi Cal Milk	A
Magnolia Omega Plus Low Fat Hi Cal Chocolate Milk	A
NESCAFE Milk Coffee Latte Can	E
Yeo’s Chrysanthemum and Luo Han Guo Tea	B
Gingen 100% Ginger Natural Beverage	A
F&N Magnolia Fresh UHT Milk	A
F&N Farmhouse Low Fat UHT Milk	E
Justin Metcalf Signature Blend Coffee Capsules	A
Kickapoo Joy Juice	A
Pokka Honey Lemon	D
Dutch Mill Yoghurt Drink with Strawberry Juice	E
Coca-Cola Life	E
Marigold UHT Strawberry Milk	A
Marigold UHT Chocolate Milk	B
Pauls Strawberry Milk	E
Coca-Cola Cherry Coca-Cola	B
Coca-Cola Diet Coke Caffeine Free	A
Marigold 100% Tropical Juice	D
Hsiang Yuan Black Sesame and Oats Powder	A
Ovaltine Jar Malted Milk	A
Pauls UHT Pure Milk	E
Pauls UHT Low Fat Milk	E
Ceres Ruby Grapefruit 100% Juice	E
Marigold 100% Pear Mixed Berries Juice	D
Pauls UHT Skimmed Milk	E
Coca-Cola Life	E
Dutch Lady UHT Strawberry Milk	E
Dutch Lady UHT Chocolate Milk	E
Pauls UHT Skimmed Milk	E
Just Juice Apple Juice	E
The Berry Company Goji Berry	E
UFC Refresh 100% Natural Coconut Water	B
Pauls UHT High Cal Low Fat Milk	E
Pauls UHT Pure Milk	E
Schweppes Slimline Indian Tonic Water	A
Tipco 100% Broccoli and Mixed Fruit Juice	E
Suntory Boss Coffee Zeitaku No Bitoh	E
Accelerade Orange (2.06 Lb.)	E
Effect Energy Drink—Carton	E
Gatorade Drink Mix Sport Drink Fruit Punch	E
Gatorade Drink Mix Sport Drink Lemon-Lime	E
Gatorade G2 Sport Drink Blueberry Pomegranate	E
Lotte Chilsung Hot 6 Energy Energy Drink	A
Lotte Chilsung Hot6 Grapefruit Energy Drink	A
Lucozade Energy Original	E
Monster Absolute Zero	A
Monster Energy Ultra Sugar Free Energy Drink	A
100 Plus Lemon Lime Isotonic Drink	E
Boost Jar	E
YOUC1000 Vitamin Lemon Health Drink	E
YOUC1000 Vitamin Orange Health Drink	E
Pinnacle Isotonic Drink—Zest	E
Pinnacle Isotonic Drink—Zest 330mL × 24 Packs	E
Naughty G Energy Drink	E
Lucozade Energy Orange	E
Lucozade Sport Isotonic Drink—Lemon	E
Monster Khaos Energy and Juice Drink	E
Monster Energy Drink	E
100 Plus Isotonic Drink	B
YOUC1000 Orange Water	E
YOUC1000 Lemon Water	E
Pocari Sweat ION Supply Drink	E
100 Plus Edge Isotonic Drink	E
Pocari Sweat ION Supply Drink (similar to 4891392000589)	E
Noble Natural Elite Drink (only sell in carton)	A
H-TWO-O Original Isotonic Drink	E
Fentimans Rose Lemonade	E
Asahi Mitsuya Cider Bottle	A
Bickford & Sons Mixer Bitter Lemon	E
Bickford & Sons Mixer Ginger Ale	E
Bickford & Sons Mixer Soda Water	A
Bickford Premium Sparkling Apple Davidson Plum	E
Bickford Premium Sparkling Red Grape with Hibiscus	E
Bickford Traditional Soda Sarsaparilla	E
Bickford Traditional Soda Creamy Soda	E
Green Spot California Orange Carbonated Drink	E
Fanta Strawberry	E
San Pellegrino Pompelmo Sparkling Beverage	E
San Pellegrino Aranciata Rossa Sparkling Beverage	E
A&W Sarsaparilla Root Beer	E
A&W Cream Soda Carbonated Soft Drink	E
Bundaberg Blood Orange	E
Bundaberg Ginger Beer	E
Bundaberg Root Beer	E
Bundaberg Pink Grapefruit	E
Dr Pepper	E
Dr Pepper Reg	E
Dr Pepper Zero	A
Virgils Flying Cauldron—Butterscotch Beer	E
Lotte Chilsung Cider Soda Lemon-Lime Soda Water	E
Sprite Sparkling Lemon-Lime Less Sugar	E
Ribena Blackcurrant No Added Sugar	E
Ribena Blackcurrant and Apple Juice Cordial (Ribena Less Sweet Blackcurrant Juice Cordial)	D
Robinsons Apple and Blackcurrant No Added Sugar	E
Robinsons Orange No Added Sugar	E
Rose Lime Juice Cordial	E
Belvoir Organic Elderflower Cordial	E
Belvoir Lime and Lemongrass Cordial	E
Belvoir Organic Ginger Cordial	E
Belvoir Raspberry and Rose Cordial	E
Nestea Lemon Blend Iced Tea	E
Crystal Light Pink Lemonade Drink Mix	A
Crystal Light Lemonade Drink Mix	A
Crystal Light Decaf Iced Lemon Tea Drink Mix	A
Pai Chia Chen Mulberry Fruit Vinegar	A
O Health Organic Black Fungus Collagen Drink	E
Dragon Brand Bird’s Nest Beverage Reduced Sugar	A
Dragon Brand Bird’s Nest Beverage Regular	A
TTL Anka Malz Drink Health Drink	A
Provitamil Oat Drink	E
MILO NUTRI G Chocolate Malt Drink	A
Pokka Premium Milk Coffee	E
Pokka Nanyang Coffee	E
NESCAFE Milk Coffee Mocha Can	E
NESCAFE SMOOVLATTE Coffee	E
NESCAFE Milk Coffee Latte Can	E
Mr. Brown Premium Iced Coffee	E
Pokka No Sugar Oolong Tea	A
Pokka Premium Afternoon Red Tea	E
Pokka Ice Peach Tea	B
Marigold Less Sweet Chrysanthemum Tea	B
Arizona Green Tea with Ginseng and Honey	C
Arizona Sweet Tea	D
Arizona Raspberry Tea	E
NutriWell Water Chestnut and Sugar Cane	E
NutriWell Chrysanthemum with Wolfberry	E
Marigold Winter Melon Tea	E
Marigold Longan Red Dates Drink	E
Oishi Honey Lemon	E
F&N Seasons Ice Lemon Tea	A
F&N Seasons Ice Peach Tea	A
Yeo’s Wintermelon Tea	E
Snapple All Natural Peach Tea	D
Sosro Tehbotol	E
Sosro Apple Fruit Tea	E
MineShine Milk Tea Drink	A
Marigold Uji Cha Momo Green Tea	A
First Brew Ginseng Chrysanthemum Tea	B
First Brew Luo Han Chrysanthemum Tea	B
T.Grand Assam Milk Tea Green	E
T.Grand Assam Milk Tea—Original	E
Alain Milliat Carrot Juice	E
Alain Milliat Red Tomato Juice	E
Allswell Longan and Red Date Drink	E
Allswell Plum Juice Drink	E
Allswell Water Chestnut and Sugar Cane Drink	B
Aquacoco Coconut Water 330 mL	E
Belle France Lemonade	E
Beutelsbacher Beetroot Juice	E
Beutelsbacher Elderberry Juice	E
Beutelsbacher Lemon Juice	E
Beutelsbacher Mt. Cranberry Juice	E
Biotta Organic Beetroot Juice	E
Ocean Spray Cranberry Light	E
Coca-Cola Light	B
East Imperial Mombasa Ginger Beer	E
Gatorade G2 Sport Drink Fruit Punch	E
Gatorade G2 Sport Drink Lemon-Lime	E
Gatorade G2 Sport Drink Orange	E
John Crabbie’s Cloudy Ginger Beer	E
Leezen Organic Veggie Fruit Enzyme Concentrate	A
Pinnacle Isotonic Drink—Tangerine	E
Glaceau XXX Acai-Blueberry-Pomegranate Vitamin Water	A
Glaceau Essential Orange-Orange Vitamin Water	A
Glaceau Power-C Dragonfruit Vitamin Water	A
Glaceau Restore Fruit Punch Vitamin Water	A
Pinnacle Science Laboratories Muscle Builder High Protein Drink—Ice Vanilla	E
Pinnacle Science Laboratories Muscle Builder High Protein Drink—Ice Belgium Chocolate	E
Pinnacle Science Laboratories Muscle Builder High Protein Drink—Ice Cappuccino	E
Suntory Iyemon Special Green Tea	A
Suntory Oolong Tea	A
Arizona Arnold Palmer Lemonade and Iced Tea	E
Reed’s Extra Ginger Brew Bottle	E
First Brew Prunella Tea	E
First Brew Cane Barley	C
First Brew Water Chestnut	B
Jia Jia Less Sugar Herbal Tea	E
F&N Seasons Reduced Sugar Grass Jelly Drink	E
Reed’s Spiced Apple Brew Bottle	E
Schweppes Dry Ginger Ale	E
MILO Peng Nutri Up Chocolate Malt Drink	E
Yakult Assorted Cultured Milk	E
Marigold 0% Fat Yoghurt Drink—Strawberry	B
Meiji Bulgaria Low Fat Yoghurt Drink	E
Cowhead Full Cream Fresh Milk	E
Meiji Strawberry Flavour Milk	E
Lipton Yellow Label Tea	A
Twinings Lemon and Ginger Tea	A
Basilur Tea Earl Grey Loose Leaf Tea	A
Twinings Lady Grey Tea	A
MILO ACTIV-GO 3 In 1 Powder	E
NESCAFE Gold Blend Decaf Instant Soluble Coffee 100 g	A
NESCAFE Ipoh White Coffee Gao Siew Dai 15S	A
Old Town 3-In-1 Classic White Coffee Mix	E
Owl Kopitiam Roast and Ground—Kopi C Kosong Coffee	E
Ceres Apple 100% Juice	E
F&N Rose Syrup Cordial	E
Lotte Chilsung Let’s Be Mocha Latte	A
Oishi Kabusecha Japanese No Sugar Green Tea	A
Heaven and Earth Jasmine Green Tea	A
Pocari Sweat ION Supply Drink	E
Red Bull Energy Drink	B
Nescafe Milk Coffee Original Can—Carton (5 + 1)	E
Starbucks Frappuccino Mocha Chilled Coffee Drink	E
Starbucks Frappuccino Chilled Coffee Drink	E
Georgia Cafe Au Lait Cold Brew	A
Heaven and Earth Ayataka No Sugar Japanese Green Tea	A
Pokka Jasmine Green Tea	A
Pokka No Sugar Japanese Green Tea	A
Pokka Premium Milk Tea	E
Asian Story Chrysanthemum Tea (Less Sugar)	B
Pokka Lemon Tea	A
Yeo’s Chrysanthemum Tea	B
Pokka Chrysanthemum White Tea	B
Marigold Uji Cha—Yuzu Green Tea	B
Yeo’s Chrysanthemum Tea	B
NutriWell Barley	E
NutriWell Lemongrass with Ginger	E
Marigold 100% Apple Juice	D
CoCoWater Pure Coconut Water—Case	E
Coco Life Coconut Water—Case	B
UFC Refresh 100% Natural Coconut Water—Case	E
UFC Refresh 100% Natural Coconut Water—Case	E
Ceres Apple 100% Juice	E
Marigold Peel Fresh Juice Drink—Tropical Mango	B
Sunkist Apple Juice	E
CoCoWater Pure Coconut Water	E
Sunkist Orange Juice	C
Marigold Peel Fresh Juice Drink—Orange	B
Florida’s Natural Ruby Red Grapefruit Juice	E
Marigold 100% Orange Juice	C
COCOLOCO Organic Coconut Water —Chilled	B
Marigold Peel Fresh Juice Drink—Apple Aloe Vera	B
Vita Coco Natural Coconut Water	B
Marigold Peel Fresh No Sugar Added Juice Drink—Apple	B
Marigold Peel Fresh Select Juice Drink–Yuzu	B
Florida’s Natural Lemonade	E
F&N Fruit Tree Fresh Apple—No Added Sugar	E
Ceres Cranberry and Kiwi 100% Juice	E
CoCoWater Pure Coconut Water	E
Wong Coco All Natural Coconut Juice with Pulp—Case	C
Marigold Peel Fresh Juice Drink—Pink Guava	B
CocoMax 100% Coconut Water—Case	B
Pepsi	A
Schweppes Dry Ginger Ale Pet Bottle	E
Coca-Cola Regular Twin Pack	E
Coca-Cola Zero Sugar	A
Schweppes Tonic Water	E
Schweppes Soda Water	A
Coca-Cola	E
Pokka Sparklin Fuji Apple Juice—Case	E
Fanta Orange	E
Orangina Zero 6 Pack	E
F&N Club Soda Water—325 mL	A
F&N Club Soda Water	A
F&N Cool Ice Cream Soda Sparkling Flavoured Drink	D
F&N Sarsi Sparkling Flavoured Drink	B
Yeo’s JusCool Sparkling Apple Juice Drink	C
Yeo’s JusCool Sparkling Peach Juice Drink	C
F&N Outrageous Orange Sparkling Flavoured Drink	D
F&N Cheeky Cherryade Sparkling Flavoured Drink	D
Yeo’s JusCool Sparkling Grape Juice Drink	C
Delamere UHT Whole Goats Milk—Sample	E
Farmhouse Fresh UHT Milk—Case	A
Cowhead UHT Pure Milk—Case	E
Lactel UHT Semi-Skimmed Milk	A
Marigold Full Cream UHT Milk	B
NESTLE Just Milk—Full Cream Milk	A
Pauls UHT Pure Milk—Case	E
Marigold Full Cream UHT Milk—Case	A
Dutch Lady UHT Full Cream Milk	A
Marigold Low Fat UHT Milk	B
F&N Farmhouse Low Fat UHT Milk—Case	E
Living Planet Low Fat Organic Dairy Milk	E
Blue Diamond Almond Breeze Unsweetened	A
Marigold Full Cream UHT Milk—Case	A
Marigold Low Fat UHT Milk—Case	A
Pauls Zymil Lactose Free Full Cream Milk	E
Pauls UHT Skimmed Milk—Case	E
Marigold UHT Australian Milk	B
Anlene Movemax Gold Milk Powder	E
Meadow Fresh Full Cream New Zealand Pure Milk	E
Meiji Fresh Milk	A
Farmhouse Fresh Milk	E
Pura Fresh Milk	E
Australia’s Own Organic Almond Milk Gluten Free UHT	E
Australia’s Own Organic Unsweetened Almond Milk	E
Pacific Natural Foods Organic Unsweetened Low-Fat Almond Non-Dairy Beverage	A
Blue Diamond Original Unsweetened Almond Breeze	A
Australia’s Own Almond and Coconut Milk UHT	E
Oatly Organic Oat Drink	B
Pacific Natural Foods Original All Natural Hazelnut Non-Dairy Beverage	E
Oatly Chocolate Oat Drink	B
Blue Diamond Barista Almond Breeze	E
Rude Health Organic Gluten Free Almond Drink	E
Rude Health Ultimate Organic Almond Drink	A
Freedom Foods Rice Milk Regular—Gluten Free Lactose Free Soy Free UHT	E
Blue Diamond Chocolate Almond Breeze	E
Blue Diamond Chocolate Unsweetened Almond Breeze	A
Blue Diamond Almond Breeze Vanilla Flavor	A
Vitasoy Original Soya Bean Drink	B
Australia’s Own Unsweetened Soy Milk	E
Pacific Natural Foods Soy Barista Series	E
Pacific Organic Soy Unsweetened Original Non-Dairy Beverage	A
Vitasoy Chocolate Flavored Soy Drink	B
Marigold Soya Bean Drink	B
Vitasoy HK Style Milk Tea	E
Vitamilk Soy Milk	A
V-Soy Multi Grain Soy Milk	E
Marigold Soya Bean Drink—Case	B
Vitasoy Original Soya Bean Drink	B
Nutrisoy with Calcium and Reduced Sugar Soya Milk	E
Yeo’s Soya Bean Drink	B
Freedom Foods Extra Milky Soy Milk Gluten Free UHT	E
Asian Story Soya Bean Milk	A
Soy Dream Organic Enriched Soy Drink	E
Unisoy Black Soy Milk Powder	A
Health Paradise Instant Soya Milk Powder NSA	E
Health Paradise Instant Black Soya Milk Powder NSA	E
Marigold Chocolate UHT Milk	B
Marigold Strawberry UHT Milk	A
Anlene Concentrate—Vanilla Hi-Calcium Milk	A
Dutch Lady UHT Chocolate Milk	E
Pacific Natural Foods Chocolate All Natural Hazelnut Non-Dairy Beverage	E
F&N Magnolia Smoo Chocolate Flavoured Milk	A
Rude Health Organic Gluten Free Coconut Drink	E
Marigold Strawberry UHT Milk—Case	A
Ensure Vanilla Milk Powder FREE Sports Bag	A
F&N Magnolia Smoo Vanilla Flavoured Milk	A
F&N Magnolia Smoo Strawberry Flavoured Milk	A
Greenfields Chocomalt Milk	E
Marigold HL Milk—Chocolate	A
Meiji Melon Flavored Milk	E
Marigold HL Milk—Strawberry	A
Meiji Chocolate Flavour Milk	E
Marigold HL Milk—Banana	A
Marigold HL Milk—Chocolate	A
Meiji Strawberry Flavour Milk	E
Meiji Coffee Flavour Milk	E
Elle & Vire Yaggo Milk Drink—Strawberry	E
MILO Can Original (5 + 1)—Case	E
MILO Peng Nutri Up Chocolate Malt Drink Bottle	E
MILO UHT Chocolate Malt Packet Drink Case	E
MILO UHT Chocolate Malt Packet Drink	E
MILO UHT Chocolate Malt Packet Drink (5 + 1) Case	E
MILO Nutri G Chocolate Malt Drink	A
Yakult Probiotic Cultured Milk Drink—Light Made In UK	E
VITAGEN Less Sugar Cultured Milk Banded Twin Pack (Assorted)	E
Meiji Paigen Culture Milk Original Flavour	E
Meiji Paigen Low Sugar Cultured Milk	E
Meiji Paigen Culture Milk Blueberry Flavour	E
Meiji Paigen Culture Milk Orange Flavour	E
Meiji Paigen Strawberry Cultured Milk	E
Nomadic Mango Lassi Exotic Yoghurt Drink	A
Nomadic Apple and Banana Oats and Yoghurt Drink	A
Emmi Swiss Yogurt Drink Strawberry	E
Marigold 0% Fat Yoghurt Drink—Mixed Berries	B
Magnolia Strawberry Yoghurt Drink	E
Marigold 0% Fat Yoghurt Drink—Mango	B
Magnolia Mango Yoghurt Drink	E
Marigold 0% Fat Yoghurt Drink - Fruit and Vegetables with Wheatgrass	E
Nestle Bliss Plus Apple Cranberry Pomegranate Yoghurt Drink	E
Nestle Bliss Plus Apple Passion Fruit Lemon Yoghurt Drink	E
Sunquick Orange Squash Concentrate	A
CJ Vinegar Drink Pomegranate	E
Monin Grenadine Syrup	A
Teisseire Grenadine Syrup	A
CJ Vinegar Drink Blueberry	E
Teisseire Green Mint Syrup	A
Teisseire Grenadine Syrup Value Size	E
Monin Lychee Syrup	A
Teisseire Lemon No Added Sugar Syrup with Stevia	E
Teisseire Green Mint Syrup Value Size	E
Gold Kili Instant Honey Chrysanthemum Drink	E
Ribena Blackcurrant	E
Bottlegreen Elderflower Cordial	E
Rose Brand Rose Flavour Syrup	A
Ocean Spray Cranberry Cocktail Juice	E
Bickford Diet Lime Cordial	E
Bottlegreen Ginger and Lemongrass	E
V8 Original Bloody Mary Mix	E
Bickford Lemon Barley Cordial	E
Bickford Lime Juice Cordial	E
Bickford Diet Lemon Cordial	E
CJ Vinegar Drink Lemon and Citron	E
Bickford Ginger Beer Cordial	E
PULCO Citron-Lemon No Sugar Added Concentrates Cordial	E
Pulco Orange Cordial	E
Bickford Lemon Lime Cordial	E
Manna Health Mix	E
Red Bull Energy Drink Sugar Free	B
Red Bull Energy Tropical Edition	A
Sultan Power Drink 250 mL	E
Sultan Cola Classic 250 mL	A
Aquarius Isotonic Drink	B
Barr-Irn Bru Sugar Free	A
Barr-Irn Bru	E
Lucozade Energy Pink Lemonade	E
Barr Irn-Bru	E
Twinings Finest Ceylon Tea 25’s	A
Twinings Lemon and Ginger Gift Pack with Lotus Biscuit	A
Super 3-in-1 Original Milk Tea	E
Twinings Pure Peppermint Tea	A
PG Tips 40S Pyramid Teabags	E
OSK Japanese Green Tea with Brown Rice	A
Lipton Teh Tarik Milk Tea Latte	E
Dilmah Camomile Tea	A
Twinings English Breakfast Tea	A
Teapigs Chamomile Flowers 50 Per Pack	A
Twinings Camomile Tea	A
Premium Matcha Green Tea	A
Twinings Earl Grey Tea	A
Dilmah English Breakfast Tea	A
Twinings Camomile and Honey Tea	A
Traditional Medicinals Organic Mother’s Milk Tea	A
Clipper Fairtrade Organic English Breakfast Tea	A
Dilmah Earl Grey Tea	A
Lipton English Breakfast Black Tea	A
NESCAFE Gold BLEND 3 in 1 15S Instant Coffee	A
Gold Roast Kopi-O Ground Coffee with Sugar	B
Cafe21 Low Fat 2 In1 Instant Coffeemix	A
NESCAFE Original 3 In 1 Instant Coffee	A
Super Essenso MicroGround Coffee—2 In 1 Coffee and Creamer	E
Super Essenso MicroGround Black (Columbian) Coffee	A
NESCAFE 25%-Percent Less Sugar Instant Coffee	E
NESCAFE Dolce Gusto Cafe Au Lait Capsules 16S	A
Moccona Continental Gold Coffee	A
NESCAFE Dolce Gusto Grande Intenso Capsules 16S	A
NESCAFE Dolce Gusto Cappuccino Capsules 8S/8S	D
NESCAFE Zero Sugar Added 2 In 1 Instant Coffee 35S	A
NESCAFE CLASSIC Jar Instant Soluble Coffee 200 g	A
NESCAFE Dolce Gusto Lungo Capsules 16S	A
Cafedirect Americano Nespresso Compatible Capsules	A
Old Town 3-In-1 Less Sugar	E
NESCAFE Gold BLEND 3 in 1 15S Instant Coffee	A
Illy Espresso Medium Roast Ground Coffee	A
Cafe21 2 in 1 Instant Coffeemix	A
NESCAFE Singapore White Coffee Gao Siew Dai Hazelnut 15S	A
NESCAFE Kopi-O Instant Coffee	A
Cafedirect Fairtrade Sao Tome 3-in-1 Drinking Chocolate	E
Cafedirect Fairtrade San Cristobal 2-in-1 Drinking Chocolate	E
MILO ACTIV-GO Regular Powder Refill Pack	E
MILO Australian Recipe Powder Refill	E
MILO ACTIV-GO Regular Powder Tin	E
MILO Australian Recipe Powder Tin	E
MILO ACTIV-GO Regular Powder Refill Pack	E
Hershey’s Natural Unsweetened Cocoa Powder	A
MILO ACTIV-GO GAO SIEW DAI	E
MILO 2 In 1 ACTIV-GO Calcium Plus Powder	E
NESTUM All Family Cereal Original	E
MILO Ice Energy Refill Pack	E
Horlicks Original	E
Green and Black’S Organic Fairtrade Cocoa Powder	A
Cadbury Fairtrade Drinking Hot Chocolate	E
NESTLE OMEGA Plus ActiCol Milk with Oats	E
Van Houten Cocoa Powder	A
MILO NUTRI G Powder	E
Ovaltine Power 10 Chocolate Flavour Malt Drink	E
Swiss Miss Dark Chocolate Sensation Hot Cocoa Mix	E
Caotina Original Chocolate Drink	E
Swiss Miss Cocoa Mix Marshmallow Madness	E
Schweppes Soda Water—Case	A
F&N Club Soda Water—Case	A
A&W Cream Soda—Case	E
Lotte Chilsung Milkis Yogurt Soda Carbonated Soft Drinks	E
Red Bull Energy Drink	B
Ice Cool Booster Energy Drink (Non-Carbonated)	E
100 Plus Isotonic Drink—Case	B
100 Plus Isotonic Drink	B
Pocari Sweat ION Supply Drink—Case	E
H-TWO-O Original Isotonic Drink—Case	A
H-TWO-O Original Isotonic Drink	A
Pai Chia Chen RTD Apple Vinegar	E
Pai Chia Chen RTD Plum Vinegar	E
Nescafe Milk Coffee Mocha Can—Carton (5 + 1)	E
Lotte Chilsung Let’s Be Coffee	E
Kiriman Blend No Sugar Ice Coffee	A
Suntory Boss Black Coffee	E
Nescafe Milk Coffee Latte Can—Carton (5 + 1)	E
Pokka Jasmine Green Tea Case (6000 mL)	A
Marigold Less Sweet Chrysanthemum Tea—Case	B
F&N Seasons Ice Lemon Tea—Case	E
Pokka Jasmine Green Tea Case (7920 mL)	A
Yeo’s Chrysanthemum Tea—Case	B
Heaven and Earth Ayataka No Sugar Japanese Green Tea—Case	A
Pokka Lemon Tea Case	E
Lotte Chilsung Corn Silk Tea	A
Marigold Less Sweet Lemon Barley	B
MILO ACTIV-GO GAO SIEW DAI 15 × 33 G	E
NESTUM 3 In 1 Cereal Drink Original	E
MILO ACTIV-GO Regular Powder Refill Pack (900 G + 100 G)	E
Twinings Jasmine Green Tea	A
Green Pot Tea Matcha Green Tea Powder	A
Twinings English Breakfast Tea 25’S	A
Clipper Organic Infusion Sleep Easy Tea	A
Bickford Lemon Juice Cordial	E
Bickford Blackcurrant Cordial	E
Teisseire Lemon Syrup	E
Belle France Grenadine Cordial	E
Pulco Lemon Cordial	E
Florida’s Natural Apple Juice	E
Marigold Apple Juice Drink (1500 mL)	C
Marigold 100% Apple Grape Juice	D
Cawston Press Cloudy Apple Juice	B
Marigold Apple 100% Juice	D
F&N Fruit Tree Fresh Apple and Aloe Vera Juice	E
Knudsen Organic Apple Juice	E
Marigold Peel Fresh No Sugar Added Juice Drink—Cranberry and Apple	B
Marigold 100% Apple Juice—Case	D
Ceres Apple 100% Juice—Case	E
Marigold Apple Juice Drink (6000mL)	C
Zico Coconut Water	B
Ice Cool Pure Coconut Water	B
Nectar Peach	A
Sapporo Nectar Sour (1400 mL)	A
Sapporo Nectar Sour (8400 mL)	A
Gina Mango Nectar	E
7D Mango Nectar Drink	E
HiPP Organic Plum Nectar Juice	E
Alain Milliat Apricot Nectar	E
Agrilife Coconut Flower Nectar (270 g)	E
Agrilife Coconut Flower Nectar (470 g)	E
Alain Milliat White Peach Nectar	E
The Coconut Company Organic Coconut Nectar Sugar	A
Alain Milliat Strawberry Nectar	E
Meiji Bulgaria Royal Fuji Apple Yoghurt Drink	E
Meiji Bulgaria Wild Berry Yoghurt Drink	E
Marigold 0% Fat Yoghurt Drink—Natural	B
Danone Actimel Multifruit Yoghurt Drink	E
Chobani Strawberry Banana Low-Fat Greek Yoghurt Drink	E
Chobani Mixed Berries Low-Fat Greek Yoghurt Drink	E
Danone Actimel Citrus and Coconut Yoghurt Drink	E
Chobani Mango Low-Fat Greek Yoghurt Drink	E
Meiji Bulgaria Golden Honey Yoghurt Drink	E
J3 Cold Pressed Juice No Sugar Homemade Yogurt	A
Nestle Bliss Plus Apple Prune Red Beet Yoghurt Drink	E
J3 Cold Pressed Juice Y4 Honeydew Yogurt Drink	A
Danone Actimel Blueberry Yoghurt Drink	E
J3 Cold Pressed Juice Y2 Red Dragon Fruit Yogurt Drink	A
J3 Cold Pressed Juice Y1 Beetroot Yogurt Drink	A
J3 Cold Pressed Juice Y3 Banana Yogurt Drink	A
Chobani Pina Colada Low-Fat Greek Yoghurt Drink	E
Magnolia Fresh Chocolate Milk	B
Vitasoy Soy Milky Lite	B
Vitasoy Soy Milky Chocolate	B
Marigold Powerbeans Fresh Soya Milk—Almond	B
Marusan Soybean Milk with Matcha	A
Marusan Soybean Milk Original	A
Marusan Soybean Milk Sesame Flavoured 50-Percentage Reduced Calories	A
Marusan Soybean Milk 45-Percentage Reduced Calories	A
Organic Valley Pasteurised Milk Whole Homo Milk Quart	B
Pauls Organic Full Cream Milk (Unhomogenised)	E
Wild Harvest Organic Whole Milk	E
Stremicks Heritage Organic Milk Vitamin D	E
A2 Whole Fresh Milk	E
Delamere Goats Milk Whole Fresh Pasteurized	E
Marigold HL Milk—Plain	A
Meiji Skimmed Milk	E
Meiji 4.3 Deluxe Milk	C
Pauls Low Fat Milk	E
Cowhead Low Fat Fresh Milk	E
Hsiang Yuan Black Sesame Powder	A
Hsiang Yuan Lotus Root and Sesame Mix	E
Manna Health Mix (200 g)	E
True Citrus True Lemon 100 Packets Vitamin Drinks	A
True Citrus True Orange 100 Packets Vitamin Drinks	A
Hsiang Yuan Oats and Brown Rice Soybean Powder	E
Hsiang Yuan Sweet Potato and Brown Rice Mix	E
Xongdur Sesame Cereal Drink (Sugar Free)—Pack of 10	E
GRB Badam Drink Mix	A
Honsei Instant Ice Limeade Juice	E
Honsei Insant Ice Pineapple Juice	E
Honsei Instant Ice Apple Juice	E
Honsei Instant Ice Mango Juice	E
Honsei Instant Ice Orange Juice	E
Viberi Blackcurrant Powder	E

**Table A10:** Comparison between lowest (least healthy) and highest (healthiest) quartile of shopping baskets by weighted average Nutri-Score value.

Nutrient	Quartile	Mean	CI	Mean Diff	*p*-Value
Weighted Average NS	Lowest-	2.17	2.1–2.24	2.42	0 *
	Highest-	4.59	4.54–4.64		
AHEI	Lowest-	41.97	40.76–43.19	2.04	0.01 *
	Highest-	44.01	42.91–45.11		
Sugar(g)	Lowest-	528.88	460.96–596.80	−275.38	0 *
	Highest-	253.50	200.59–306.42		
kcal	Lowest-	14828.57	13089.05–16568.1	−5260.42	0 *
	Highest-	9568.15	7716.52–11419.78		
Totalfat(g)	Lowest-	867.11	720.85–1013.38	−612.70	0 *
	Highest-	254.41	217.7–291.12		
Saturatedfat(g)	Lowest-	242.60	206.94–278.26	−160.07	0 *
	Highest-	82.53	69.45–95.60		
Sodium(mg)	Lowest-	13969.45	10729.47–17209.44	−8472.91	0 *
	Highest-	5496.54	4667.57–6325.51		
Dietaryfiber(g)	Lowest-	76.98	66.10–87.87	22.30	0.04 *
	Highest-	99.28	81.13–117.42		
Protein(g)	Lowest-	386.22	28–491.44	20.28	0.73
	Highest-	406.50	357.74–455.26		

* *p* < 0.05.
